# Cave Drip Water-Related Samples as a Natural Environment for Aromatic Hydrocarbon-Degrading Bacteria

**DOI:** 10.3390/microorganisms7020033

**Published:** 2019-01-25

**Authors:** Eric L. S. Marques, Gislaine S. Silva, João C. T. Dias, Eduardo Gross, Moara S. Costa, Rachel P. Rezende

**Affiliations:** 1 Department of Biological Sciences, State University of Santa Cruz, Rod. Jorge Amado, Km 16, Ilheus CEP 45662-900, Brazil; gislaine1206@gmail.com (G.S.S.); jctdias@uesc.br (J.C.T.D.); moara_costa@hotmail.com (M.S.C.); 2 Department of Agricultural and Environmental Science, State University of Santa Cruz, Rod. Jorge Amado, Km 16, Ilheus CEP 45662-900, Brazil; egross@uesc.br

**Keywords:** Sphingomonadales, microorganisms, *ndo* gene, naphthalene, NGS, Illumina, 16S rDNA

## Abstract

Restricted contact with the external environment has allowed the development of microbial communities adapted to the oligotrophy of caves. However, nutrients can be transported to caves by drip water and affect the microbial communities inside the cave. To evaluate the influence of aromatic compounds carried by drip water on the microbial community, two limestone caves were selected in Brazil. Drip-water-saturated and unsaturated sediment, and dripping water itself, were collected from each cave and bacterial 16S rDNA amplicon sequencing and denaturing gradient gel electrophoresis (DGGE) of naphthalene dioxygenase (*ndo*) genes were performed. Energy-dispersive X-ray spectroscopy (EDX) and atomic absorption spectroscopy (AAS) were performed to evaluate inorganic nutrients, and GC was performed to estimate aromatic compounds in the samples. The high frequency of Sphingomonadaceae in drip water samples indicates the presence of aromatic hydrocarbon-degrading bacteria. This finding was consistent with the detection of naphthalene and acenaphthene and the presence of *ndo* genes in drip-water-related samples. The aromatic compounds, aromatic hydrocarbon-degrading bacteria and 16S rDNA sequencing indicate that aromatic compounds may be one of the sources of energy and carbon to the system and the drip-water-associated bacterial community contains several potentially aromatic hydrocarbon-degrading bacteria. To the best of our knowledge, this is the first work to present compelling evidence for the presence of aromatic hydrocarbon-degrading bacteria in cave drip water.

## 1. Introduction

Caves are aphotic environments isolated to some degree from the surface and lacking photosynthetic activity [1,2,3]. Consequently, a cave environment commonly has low levels of organic matter and some essential micronutrients change according to the mineralogical composition associated with carbonate rocks [4]. However, other factors such as runoff, bats, tourism, invertebrates’ feces, and drip water could exert a significant influence on the organic nutrients available in caves [5] and, thus, on the cave microbial communities. 

Microbial communities in caves have been studied, with a focus on microbial or biofilm associations in the cave environment [4,6,7] and external contamination caused by tourism [8,9]. Despite all the studies in caves, there is a lack of information regarding the influence of dripping water and its effects on cave floor sediment microbial communities. Microorganisms in dripping water are involved in the formation of speleothems, as well as in biofilm on the walls [10,11,12,13]. Many bacteria found in caves can induce calcite precipitation [11,14]. However, dripping water could transport organic and inorganic material from the surface [15,16,17]. This material carried to caves could change the availability of nutrients and modify microbial communities in favor of a chemoheterotrophic-based community.

We hypothesize that drip water microbial communities can degrade transported organic matter such as aromatic compounds. Among those compounds, naphthalene is one of the most well studied, and it has been a model compound for aromatic metabolism [18] and is usually found in caves [16], while several other aromatic compounds are found in low concentration in soils due to the partial degradation of lignin [19]. Among the genes that encode enzymes to degrade naphthalene, the *nahAc* and *phnAc* cluster III are those with greatest environmental importance due to their presence in common soil bacteria [20] and codify the large subunit of different naphthalene dioxygenase, and those genes were used in the present study.

The presence of aromatic compounds and aromatic hydrocarbon-degrading bacteria in caves is partially unknown with several studies having aimed to detect such compounds in karstic aquifer [21,22], but no relation with drip water was analyzed. Besides alluvial transportation, the presence of aromatic compounds in cave air has also been demonstrated as an alternative transport mechanism for such compounds [23]. 

This paper aims to analyze the occurrence of potential bacterial degradation of aromatic compounds in the bacterial communities of dripping water, and drip-water-saturated and unsaturated sediment samples from two different carbonate caves.

## 2. Materials and Methods 

### 2.1. Sampling and DNA Extraction

Two caves were selected for this study: Gruta do Bom Pastor (cave identified as GBP in the text and G associated with a sample, 10°39′05.99″ S, 37°55′26.87″ W) and Furna do Fim do Morro do Parafuso (cave identified as FFMP or F, 10°38′25.89″ S, 37°52′04.13″ W). Sampling was authorized by the Ministério do Meio Ambiente (MMA/ICMBio/SISBIO – No. 26304). These caves are located 8 kilometers from each other in Paripiranga, Bahia State, Brazil (Figures S1 and S2), a hot semi-arid location. The municipal area is based on Neoproterozoic rocks, with meta-limestone (metamorphosed carbonate rock), meta-dolomite and metapelite prevailing in the caves. Both caves are in limestone/meta-limestone hydrogeological domains that represent an aquifer system dominated by limestone, magnesian limestone, and dolomite rocks that can undergo the karstic dissolution that led to caves, sinkholes, dolines, and other erosive features related to this type of rock. The linear development of the studied caves put GBP and FFMP at 236 m and 234 m respectively. However, GBP has a considerable vertical projection for a small cave, at approximately 75 m. The first of the 3 chambers in GBP was also used as a pilgrimage destination; however, the sample sites appear preserved, especially the last chamber, which was not used in this way. No bats nor guano were visible in the sampling sites, but there are bats and guano in other chambers of both caves. Runoff occurs in GBP, and a variety of invertebrates were observed in FFMP. GBP and FFMP are located respectively 12 and 5 km from the closest city (Paripiranga), but small villages and family houses are located nearby. Above GBP is a corn plantation, while FFMP is located beneath native vegetation.

Composite sediment samples were collected from 5 subsamples in a 3 m^2^ area to obtain approximately 200 g. Dripping water was collected in sterile containers from several stalactites to reach at least 50 mL (identified as F1 and G1). The sediment samples collected were drip-water-saturated sediment (F2 and G2) and drip-water-unsaturated sediment (F3 and G3). All sediment samples were placed in sterile plastic bags and transported immediately on ice to the laboratory, where part of them was analyzed and processed for their physicochemical characteristics. For each sediment sample, the temperature (Incoterm, São Paulo, Brazil) was measured in situ, after sampling, with a thermometer in contact with the sediment.

DNA from non-sieved sediment samples was extracted using the MoBio PowerSoil^TM^ DNA kit (MoBio Laboratories Inc., Carlsbad, CA, USA) according to the manufacturer’s instructions. The kit was also used for DNA extraction from drip water cells collected on a 0.22 μm membrane.

### 2.2. Gas Chromatography Analysis

Aromatic compounds were extracted in triplicate from 10 g of soil or 10 mL of distilled water. To extract those compounds, 10 mL of hexane:acetone (1:1, v/v; FMaia LTDA, Cotia, Brazil) were added and sonicated (Ultrasonic cleaner, Maxiclean 1600, Indaiatuba, Brazil) for 30 min. After that, an additional 10 mL hexane:acetone was added, vortexed for 1 min, and sonicated again for 30 min. The extracted solution was stored in a glass vial containing Na_2_SO_4_ before analysis. Chromatographic analyses were performed on a Shimadzu gas chromatograph using an RTX-5 column (Restek, Bellefonte, PA, USA; 30 m, 0.25 mm; 0.30-µm film) with hydrogen as the carrier gas. One microliter of each sample was injected at 280 °C for 1 min. The temperature column was followed: 40 °C for 5 min followed by a heating speed of 10 °C/min up to 250 °C, hold for 20 min. A polynuclear aromatic hydrocarbons mix (Supelco, Sigma-Aldrich, St. Louis, MO, USA) was used as a standard for identification and quantification of corresponding peaks. The quantification was made in a serial dilution of standard.

### 2.3. 16S rDNA Gene Sequencing

The amplification of the 16S rRNA gene V3/V4 region was carried out using the 341F (5′-CCT ACG GGR SGC AGC AG-3′) and 806R (5′-GGA CTA CHV GGG TWT CTA AT-3′) primers [24,25]. The 16S rRNA gene libraries were constructed following a PCR-based protocol [26] and sequenced using the MiSeq Sequencing System (Illumina Inc., San Diego, CA, USA) with the V2 kit, 300 cycles. Reads were separated by barcoding and trimmed using Geneious 11.0.4. Chimeras were detected using DECIPHER [27]. Operational taxonomic units (OTUs) assigned by clustering the sequence against the Ribossomal Database Project (RDP Tools version 2.12) [28] and EZBioCloud database [29]. A cluster analysis based on the abundance of each OTU was made on PAST 3.1 software (Natural History Museum - University of Oslo, Oslo, Norway, https://folk.uio.no/ohammer/past/). These sequencing data have been submitted to the GenBank database under accession number SRP128684.

### 2.4. PCR Conditions and DGGE

The denaturing gradient gel electrophoresis (DGGE) procedure is described on Supplemental Information (Text S1). The primers amplified the *ndo* genes *nahAc* and *phnAc* [20]. Selected DGGE bands were aseptically excised and stored in Milli-Q water at 4 °C for 2 days before being put at –20 °C until re-amplification with the NAPH-2F and NAPH-2R primers [20]. After purification with the kit Purelink™ PCR (Invitrogen, São Paulo, Brazil), DNA was cloned using the TA cloning kit^®^ (Invitrogen, São Paulo, Brazil) following the manufacturer’s instructions. Transformed colonies were amplified using the M13F (5′-GTA AAA CGA CGG CCA GT-3′) and M13R (5′-CAG GAA ACA GCT ATG AC-3′) primers recommended by the cloning kit’s manufacturer. The PCR reactions contained 3 mM MgCl_2_, 1× PCR buffer, 0.2 mM of each dNTP (Promega, São Paulo, Brazil), 5 pmol of each primer, 0.05 U/µL of Taq DNA polymerase (Promega, São Paulo, Brazil), and 1.5 μL of DNA template. The PCR conditions used were 30 cycles of 94 °C for 20 s, 55 °C for 15 s and 72 °C for 60 s. The amplicons were sequenced in ABI-Prism 3100 Genetic Analyzer (Applied Biosystems, Carlsbad, CA, USA), processed, and analyzed using the Phrap/Phred/Cross Match software [30] and BLASTN and BLASTX tools [31]. Phylogenetic analysis was performed using MEGA7 software and a neighbor-joining tree with Tajima–Nei model was constructed and the topology confirmed by bootstrap analysis (*n* = 1000 replicates).

### 2.5. Physicochemical Analysis

A part of the samples was sieved through a 2-mm mesh, and 150 g of the samples was sent to the soil analysis service provided by the Department of Soil Sciences ESALQ – USP in Piracicaba, Brazil for analysis. The following parameters were determined for each soil sample: pH in 0.01 mol/L of CaCl_2_; phosphorus, potassium, calcium, and magnesium in cationic exchange resin; organic matter by dichromate/colorimetry; and potential acidity (H + Al) by the SMP pH method [32]. Energy-dispersive X-ray spectroscopy (EDX) was performed by a scanning electron microscope model Quanta 250 (FEI Company, Hillsboro, OR, USA). Sediment samples were mounted in stubs, and 3 distinct points were analyzed at 15 kV. Average and standard deviation were calculated for each sample from a triplicate analysis. Atomic absorption spectroscopy (AAS) was performed for the water samples [33].

## 3. Results

### 3.1. Gas Chromatography Analysis

Samples were analyzed for the presence of aromatic compounds. Two aromatic compounds were detected in the studied samples (Table 1). Naphthalene and acenaphthene were detected in F1 and F2 samples, while no aromatic compound was detected in the F3 sample. Only naphthalene was detected in all samples from GBP, G1, G2, and G3. The amount of identified aromatic compounds in both caves was higher in the drip water samples and lower in drip-water-unsaturated sediment (Table 1). 

### 3.2. 16S rRNA Gene Sequencing

**Figure 1:**
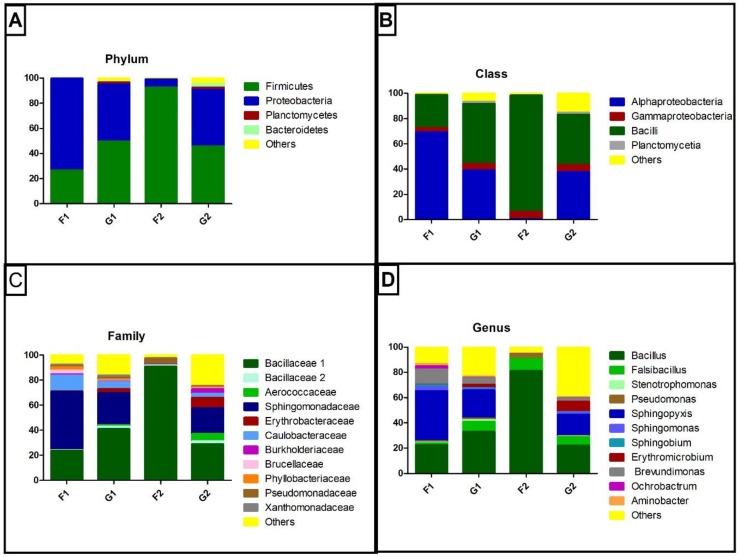
Taxonomic classification of reads for the cave samples: (**A**) phylum level, (**B**) class level, (**C**) family level and (**D**) genus level. Values in %.

To investigate potential bacteria that can use aromatic compounds and the bacterial community itself, 16S rRNA gene sequencing was performed. Illumina sequencing generates a highly variable number of reads for each sample. After processing, the 412,979 good-quality reads were distributed among four samples very unequally, ranging from 2658 (G1 and G2) to 360,138 (F2) reads per sample. The most represented phyla were determined (Figure 1A), with Proteobacteria and Firmicutes representing at least 90% of OTUs in all samples. Bacilli were the most abundant class in three samples (G1, G2, F2), and Alphaproteobacteria in one sample (F1) (Figure 1B). Alphaproteobacteria were also the dominant Proteobacteria in three of four samples (F1, G1, G2), with at least 86% representation (Figure S3), while Gammaproteobacteria dominated one sample. At family level (Figure 1C) the pattern was repeated with Bacillaceae (Bacilli, Firmicutes) and Sphingomonadaceae (Alphaproteobacteria) predominating in a similar distribution as a class. Similarly, observed at genus level, *Sphingopyxis* (Alphaproteobacteria) was the most abundant genus in one sample (F1) and the second most abundant in two samples (G1 and G2) while *Bacillus* (Bacilli) was the most abundant genus in three samples (G1, G2, F2) and the second in one sample (F1) (Figure 1D). Despite the dominance of the mentioned taxa, other taxa were also detected, such as the families Erythrobacteraceae, Burkholderiaceae, Pseudomonadaceae, Xanthomonadaceae, and Phyllobacteriaceae. 

**Figure 2:**
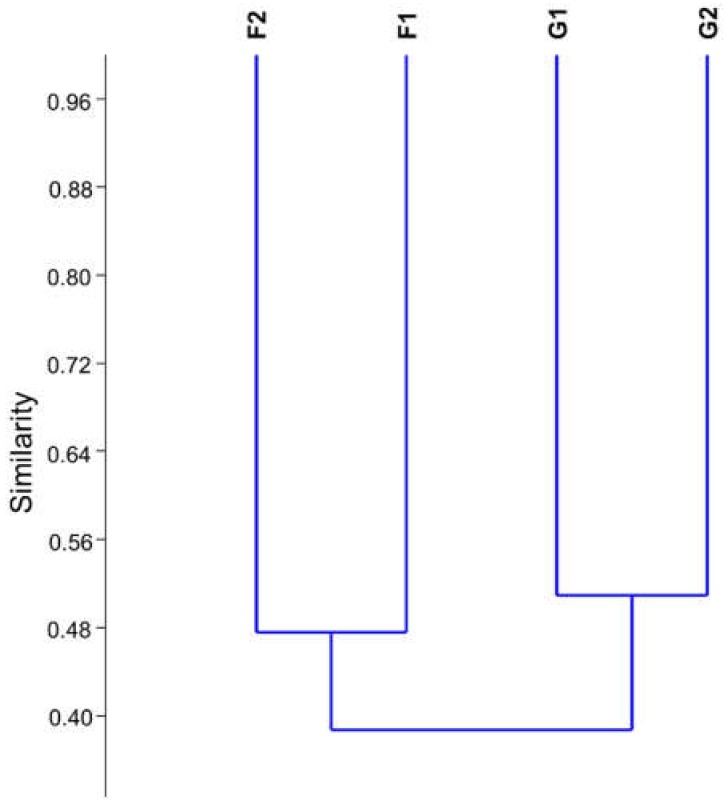
Similarity dendrogram from 16S rDNA sequencing of cave samples.

Microbial community cluster analysis based on 16S rDNA sequencing showed samples from each cave grouped with a similarity below 55% while the similarity among different caves was under 40%, corroborating DGGE data (Figure 2) of higher similarity among caves than among sample type.

### 3.3. PCR and DGGE of ndo Genes

**Figure 3:**
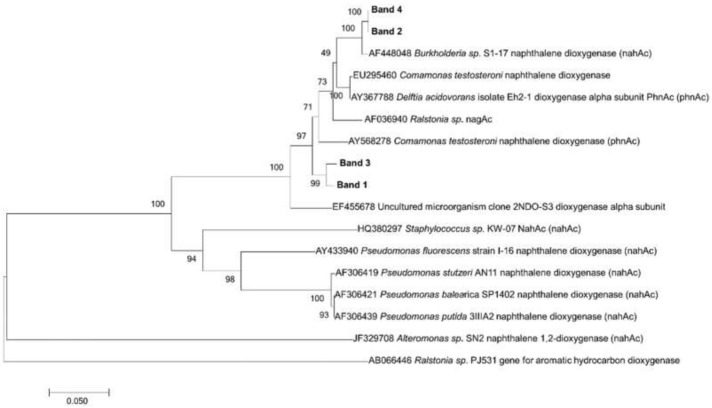
Phylogenetic affiliation of predicted naphthalene dioxygenase amino acids sequence from bands excised from denaturing gradient gel electrophoresis (DGGE) of *ndo* genes. Bootstrap values (*n* = 1000 replicates) are shown next to each branch.

Follow the detection of naphthalene and potential aromatic hydrocarbon-degrading bacteria, the presence of genes coding for naphthalene dioxygenase (*ndo* genes) was analyzed in the samples. PCR was performed, with amplification observed in four of six samples. The four samples that amplified for *ndo* genes were drip water (G1 and F1) and saturated sediment samples (G2 and F2); all six samples were subjected to DGGE analysis (Figure S4A), but no visible bands were present on the unsaturated sediment samples (G3 and F3). In a dendrogram analysis (Figure S4B) based on DGGE profiles, low similarity was observed among samples (<35%), and the saturated sediment sample (G2) presented the lowest similarity with other samples. The DNA of four bands excised from the gel were sequenced, and phylogenetic analysis showed *ndo* bands closely related to the *Burkholderia nahAc* gene from the F1 sample, as well as *nahAc* and *phnAc* from different Burkholderiales from the G1 sample (Figure 3).

### 3.4. Physicochemical Analysis

Physicochemical soil analysis (Table 2) showed an elevated level of organic matter for oligotrophic caves, phosphorus, calcium, and magnesium. Also, a low pH was measured in the drip-water-saturated sediment (G2) in comparison with the unsaturated sediment (G3), and slightly lower pH in sample saturated with drip water (F2) in contrast with unsaturated from FFMP. The drip water analysis showed a high amount of calcium and magnesium as expected for a limestone cave, a small amount of copper, and an absence of manganese, iron, and zinc (Table 3). In the EDX analysis (Table 4) other elements besides those observed in Table 3 and Table 4 were present, highlighting the presence of iron, especially in G2 and F3, with at least 10 times more iron than other samples. Sulfur was only detected in the G2 sample. Other elements were found in low amounts in at least one replicate.

## 4. Discussion

Limestone caves appear to be isolated from the surface. However, fractured rock allows water to percolate from the surface to the cave interior, creating several speleothems and the cave itself. The percolation water can transport nutrients, breaking the rock barrier that partially isolates the cave interior from a nutritional point of view [15,16,17]. Variance in the dominant bacterial community observed in DGGE showed an influence of drip water on bacterial sediment profiles (Text S1, Figure S5) confirmed by next-generation sequencing. This characteristic was better observed in GBP due to the number of stalactites and intensity of dripping, as both were visibly higher in GBP than in FFMP. The differences in the frequency of water dripping and number of stalactites (Figure S2) affect the dripping water influence on the bacterial community and could explain the proximity of FFMP sediment samples profiles in the dendrogram (Figure S5), even considering the small sampling size. Sediment humidity may explain those differences and also the physicochemical differences. However, other factors could be related to the variance in the bacterial community. Previous studies on Spanish and Chinese caves have shown that lipids and hydrocarbons could be transported by percolation water [17,34] and other compounds may explain at some degree the high level of organic matter. Although the caves are not located under dense forests, corn plantation and caatinga vegetation (Brazilian semi-arid vegetation) can provide hydrocarbons to the cave by partially decomposed surface leaves, which can be transported into the cave by percolation water. The detection of aromatic compounds in our samples provides evidence for our hypothesis that microbial communities living in environments influenced by drip water can degrade aromatic compounds. Those bacteria may use aromatic compounds as carbon or energy sources, such as the naphthalene and acenaphthene identified in the present study, and already associated with drip water in other caves [16,23,35]. The decreasing amount of aromatics in samples far from drip water indicates that the drip water is the source of such compounds. We also analyze the presence of *ndo* genes and quantification of aromatic compounds in two other caves without visible water-dripping. The *ndo* gene and aromatic compounds were only detected in the entrance and the first sample collected inside the cave (Text S2, Table S1), supporting the idea that drip water transports most aromatic compounds into the cave.

The presence of *ndo* genes is an indication that those bacteria may be using aromatic compounds as a carbon or energy source, as already described for non-cave environments [36]. The enzyme encoded by the *ndo* gene is part of the main pathway to aerobic degradation of naphthalene, however, the alpha subunit of this enzyme is highly variable among bacterial species [37] and can be part of the degradation pathway of other aromatic compounds such as the acenaphthene [35] identified in F1 and F2 samples. The absence of those genes in unsaturated samples is an indicator of the importance of *ndo* genes expression in a drip-water-influenced natural environment, also supported by the amount of naphthalene itself, as a possible selective characteristic that allows those bacteria to thrive in an environment with aromatic compounds in comparison to one without those compounds. 

The Illumina 16S rRNA gene sequencing generated a reduced number of reads in two samples due to a low amount of DNA extracted in such low-biomass samples. The difference in read count influences the diversity indices (Text S3, Table S2), however, normalized analysis showed a higher diversity in bacterial drip water samples, probably related to the low frequency of spore-forming bacteria such as *Bacillus* in the FFMP drip-water sample (F1) (Table S2). Illumina 16S rRNA gene sequencing showed a dominance of aromatic-degrading bacteria, especially the ones associated with the Proteobacteria. In particular, the family Bacillaceae was the dominant family in most of the samples and represents a metabolically diverse member of the Firmicutes that can degrade aromatic compounds [38]. Among Proteobacteria, Sphingomonadales are a common bacterium found in these caves, and are known for their ability to degrade aromatic compounds. *Sphingopyxis*, *Sphingobium*, and *Sphingomonas* represent the dominant genera of this family, and they can degrade both naphthalene and acenaphthene detected in the samples [39,40]. Other bacteria able to degrade both aromatic compounds were identified in the samples, such as the members of Burkholderiales, Pseudomonadales and Xanthomonadales, associated with the genera *Burkholderia*, *Pseudomonas* and *Stenotrophomonas*, respectively [41]. *Aminobacter*, found especially in G1 and G2, includes some species known to degrade pesticides [42], which may be related to the corn plantation above the cave. Other bacteria identified in the cave samples were also associated with aromatic hydrocarbon degradation, such as Caulobacteriales (*Brevundimonas*) and Rhizobiales (*Ochrobactrum*) [43,44]. The *ndo* gene sequencing for DGGE recovered Betaproteobacteria instead of the dominating Alphaproteobacteria from the Illumina analysis. It is possible that the primers used to amplify *ndo* genes do not amplify the Sphingomonadales genes that codify enzymes that degrade naphthalene, as it was not used as a target strain in the design of the primers [20]. The detection of Burkholderiales on DGGE of naphthalene dioxygenase gene and Illumina 16S rRNA gene sequencing corroborate a previous assumption that these bacteria carry aromatic hydrocarbon-degrading genes. However, horizontal gene transfer may play a significant role in the distribution of aromatic hydrocarbon-degrading genes [45], which could represent a limitation in the use of *ndo* sequencing to identify bacteria.

The bacterial taxa identified in the present study have also been found in other caves, including a genome of *Sphingopyxis* isolated from Cahuilla cave [46] and also identified in drip water with other genera such as *Pseudomonas*, *Bacillus*, *Stenotrophomonas*, and *Sphingomonas* [11,14,47]. However, in previously drip water studies Beta- or Gamma-proteobacteria dominated, according to the time of year [14], and not the Alphaproteobacteria and Bacilli identified in the present study. It is also important to mention that most of the microbial studies in caves are done in high latitudes where temperatures are at least 10 °C lower [48,49] than caves studied at lower latitudes, like those in the northeast of Brazil. The interface between drip water and affected sediment creates a new and possibly unique microenvironment in drip-water-saturated samples. It mixes chemical characteristics from drip water and sediment that potentially make those kinds of samples unique among other sediment or drip water samples. They also show the importance of epigean vegetation to this water-saturated-sediment cave microbiome providing evidence of the importance of preserving the natural biome above caves [50].

Based on the difference of profiles from drip water and saturated and unsaturated sediment, the presence of *ndo* genes and aromatic compounds and identification of bacteria associated with aromatic hydrocarbon degradation, it is possible to conclude that the drip-water-associated bacterial communities have several members of potential aromatic hydrocarbon-degrading bacteria. The presence of aromatic compounds seems to favor the growth and survival of such bacteria that can thrive by heterotrophic means transported by drip water, and aromatic compounds are one of the main energy and carbon sources for such communities. Further work will be carried out to analyze the degradation capability of cultured bacteria, and prospects for other genes and molecules transported by drip water, such as aliphatic compounds and lipids. 

## Figures and Tables

**Table 1:** Aromatic compounds detected in drip water and sediment from two caves.

Sample	Aromatic Compound (ng/g or ng/mL)	
	Naphthalene	Acenaphthene
**F1**	3.65 ± 0.96	1.65 ± 0.67
**F2**	2.71 ± 0.43	1.13 ± 0.48
**G1**	1.96 ± 0.32	-
**G2**	1.47 ± 0.62	-
**G3**	0.53 ± 0.15	-

*- not detected.*

**Table 2:** Physicochemical analysis of cave sediment samples.

Sample	pH	Temp °C	OM g/L	P mg/L	K mmole/L	Ca mmole/L	Mg mmole/L	H + Al mmole/L	SB	CEC	V%
GPB2	4.5	28.0	36	252	1.0	724	96	171	820.8	992.2	83
GBP3	6.5	30.0	15	4464	14.5	1070	168	17	1252.0	1269.0	99
FFMP2	6.8	23.0	81	311	9.5	430	45	9	484.5	493.9	98
FFMP3	7.0	23.5	57	533	4.1	520	24	9	548.1	556.6	98

pH in water; temp: temperature; OM: organic matter; H + Al: exchangeable acidity; SB: sum of bases; CEC: cation-exchange capacity; V: base saturation.

**Table 3:** Chemical analysis by atomic absorption spectroscopy, average of dripping frequency and pH from cave drip water samples.

Sample	pH	Drip/s	Cu mg/L	Ca mg/L	Mn mg/L	Fe mg/L	Zn mg/L	Mg mg/L
GBP1	6.1	1.1	0.015	7.353	<0.001	<0.001	<0.001	51.657
FFMP1	6.7	0.6	0.026	11.250	<0.001	<0.001	<0.001	69.598

**Table 4:** Energy-dispersive X-ray spectroscopy (EDX) analysis of sediment samples from Gruta do Bom Pastor and Furna do Fim do Morro do Parafuso. Numbers 2 and 3 in sample identification indicate respectively drip-water-saturated samples and unsaturated sediment samples. Values in % followed by standard deviation.

Sample	C	O	Fe	Mg	Al	Si	P	K	Ca	S	Co	Na	Ru	Mo
**GBP2**	31.7 ± 9.7	52.9 ± 4.9	5 ± 3.4	1.6 ± 0.9	0.66 ± 0.05	1.3 ± 0.5	2.1 ± 1.0	<0.01	1.7 ± 0.8	2.5 ± 0.8	0.9 ± 0.9	1.5 ± 0.3	<0.01	<0.01
**GBP3**	11.7 ± 2.4	58.1 ± 3.0	0.4 ± 0.3	0.91 ± 0.84	7.2 ± 3.4	10.2 ± 5.5	8.6 ± 7.1	1.6 ± 1.1	0.19 ± 0.11	<0.01	<0.01	<0.01	0.21 ± 0.12	<0.01
**FFMP2**	1.5 ± 1.5	48.5 ± 3.1	0.8 ± 0.2	1.25 ± 0.1	12.1 ± 2.5	32.3 ± 2.9	2.0 ± 1.1	1.1 ± 0.4	1.5 ± 0.6	<0.01	0.02 ± 0.02	<0.01	<0.01	<0.01
**FFMP3**	9.0 ± 3.3	48.4 ± 1.4	16.6 ± 0.4	4.1 ± 1.3	5.8 ± 0.7	13.1 ± 5.6	1.5 ± 0.6	0.05 ± 0.04	0.97 ± 0.4	<0.01	<0.01	<0.01	<0.01	0.15 ± 0.2

## Supplementary Materials

The following are available online at http://www.mdpi.com/2076-2607/7/2/33/s1, Figure S1: Geographic location of the studied caves, Figure S2: Images of the studied caves, Figure S3: DGGE analysis of *ndo* genes, Figure S4: Taxonomic classification of proteobacteria from cave samples, Figure S5: DGGE analysis of bacterial V6-V8 16S rDNA, Text S1: DGGE analysis, Text S2: Aromatic and *ndo* genes detection in other caves, Text S3: Diversity analysis, Table S1: Aromatic compounds detected along Gruta do Lapão and Gruta de Manoel Ioiô caves, Table S2: Diversity indexes of bacterial community from cave samples.
