# Supplementary material for: Cave Drip Water-Related Samples as a Natural Environment for Aromatic Hydrocarbon-Degrading Bacteria
Source: Microorganisms. 2019 Jan 25;7(2):33. doi: 10.3390/microorganisms7020033 (PMC6406655; doi:10.3390/microorganisms7020033)
Supplement: Supplementary file 1 [file microorganisms-07-00033-s001.pdf]

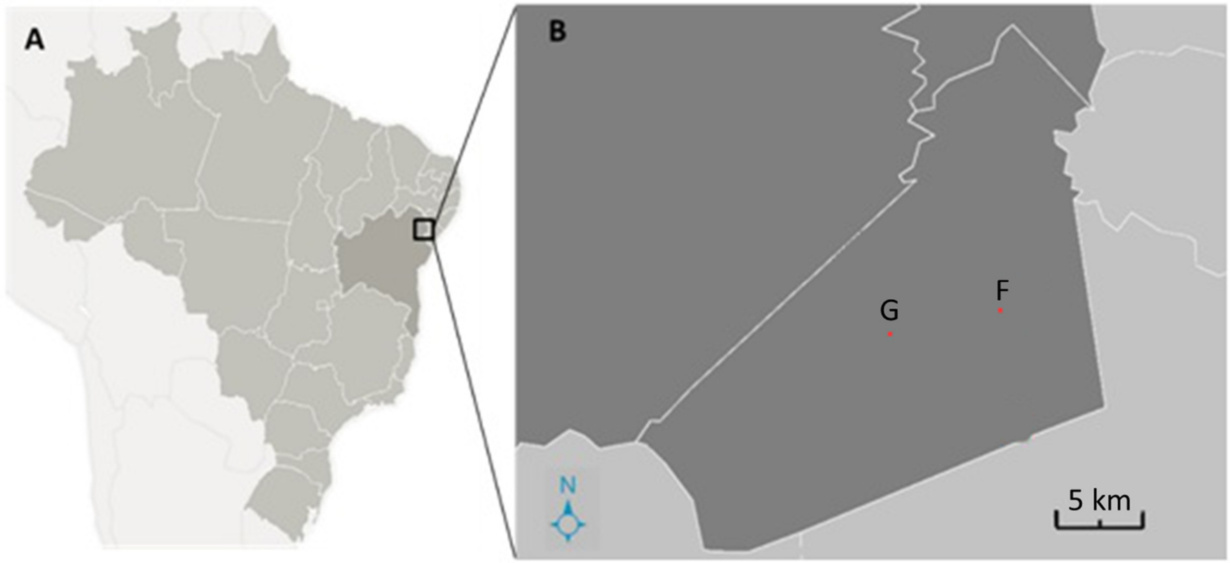

Figure S1. Geographic location of the studied caves. Map of Brazil (**A**) and Paripiranga (**B**). In dark is the state of Bahia and in light grey the other states (A).

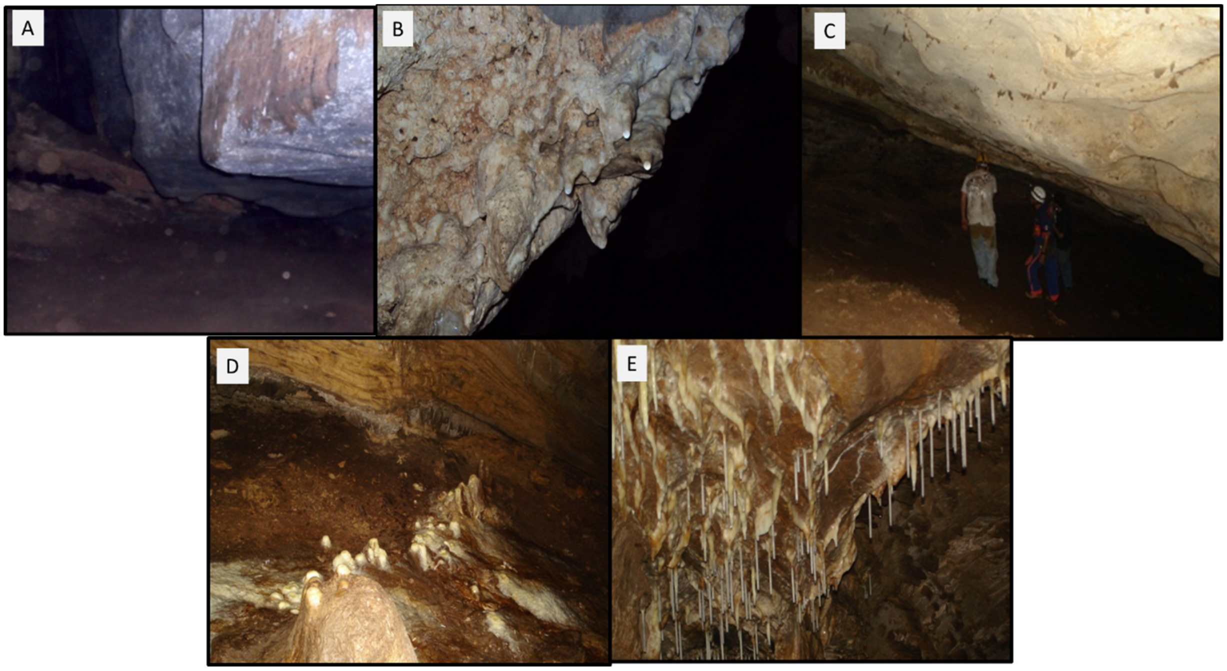

Figure S2. Images of the studied caves. In (**A**) and (**B**) Furna do Fim do Morro do Parafuso with small stalactites visible in (B) where part of sample F1 was collected. In (**C**), (**D**) and (**E**) Gruta do Bom Pastor near the G3 sampling site (C), stalagmites near G2 sampling site (D) and stalactites where sample G1 was collected (E).

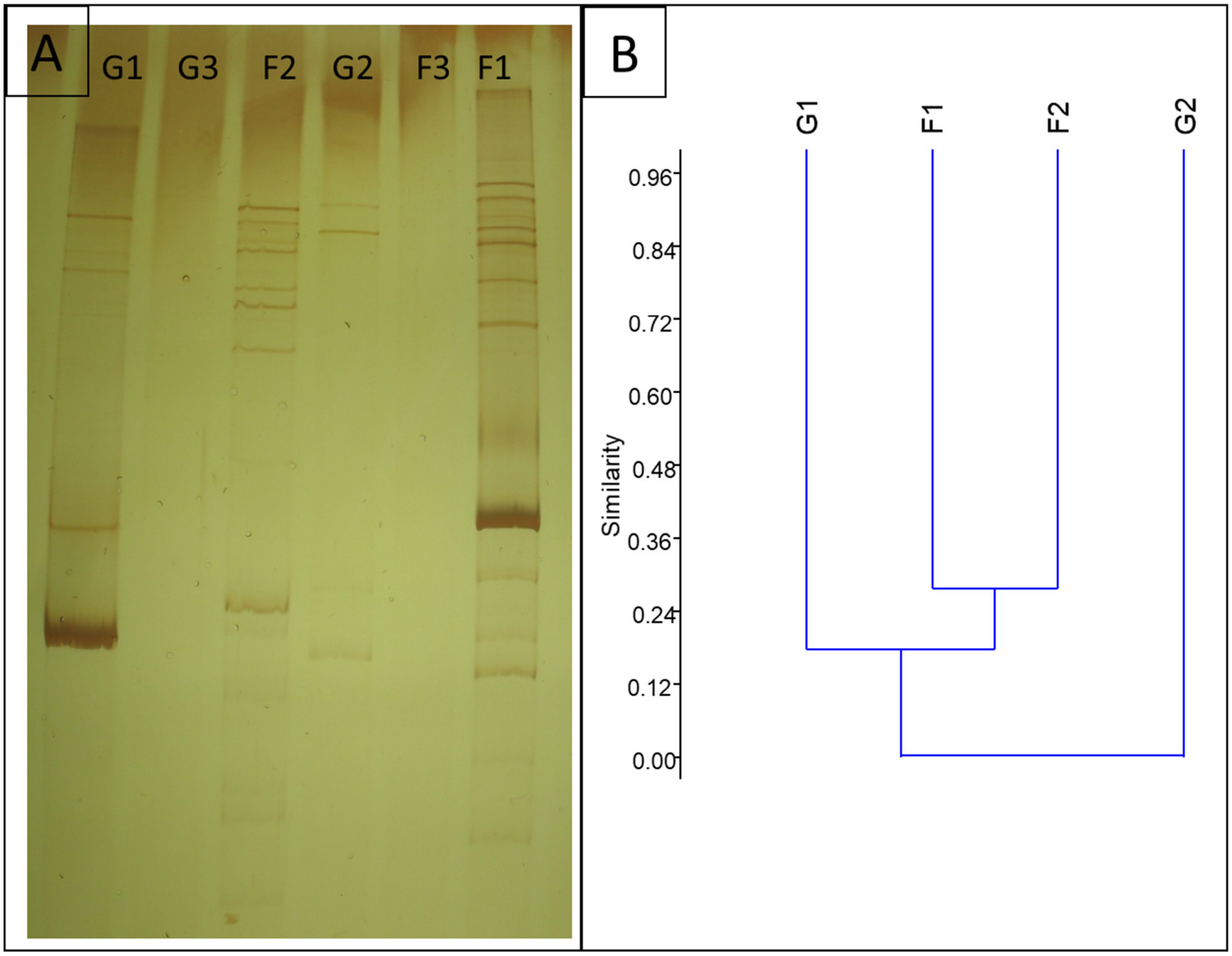

Figure S3. DGGE analysis of *ndo* genes. (**A**) DGGE image and (**B**) Jaccard dendrogram of similarity. Samples were labeled as G and F for different caves followed by "1", "2" or "3" that represent, respectively, drip water, saturated sediment with drip water and unsaturated sediment samples.

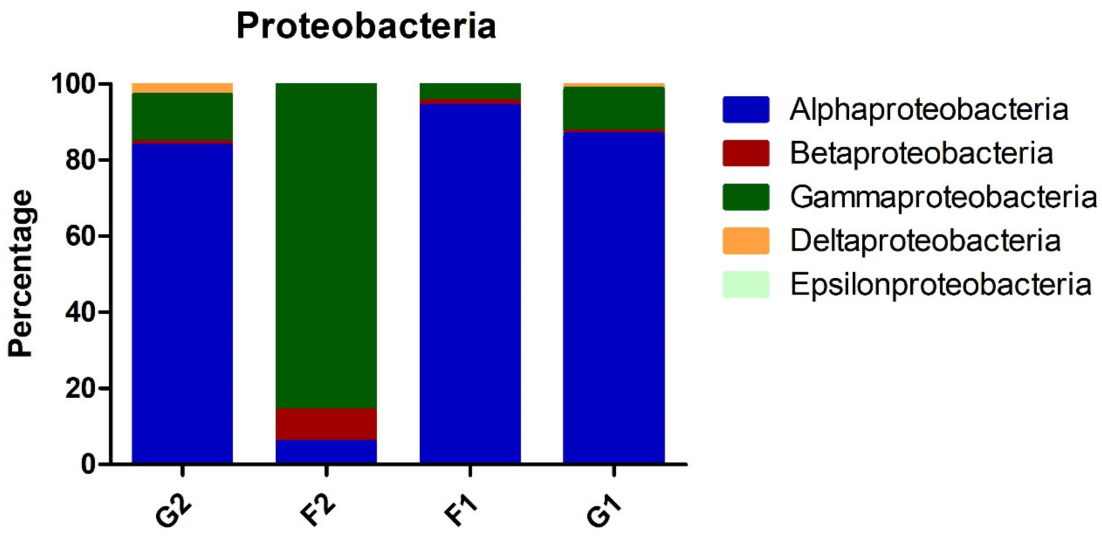

Figure S4. Taxonomic classification of proteobacteria from cave samples.

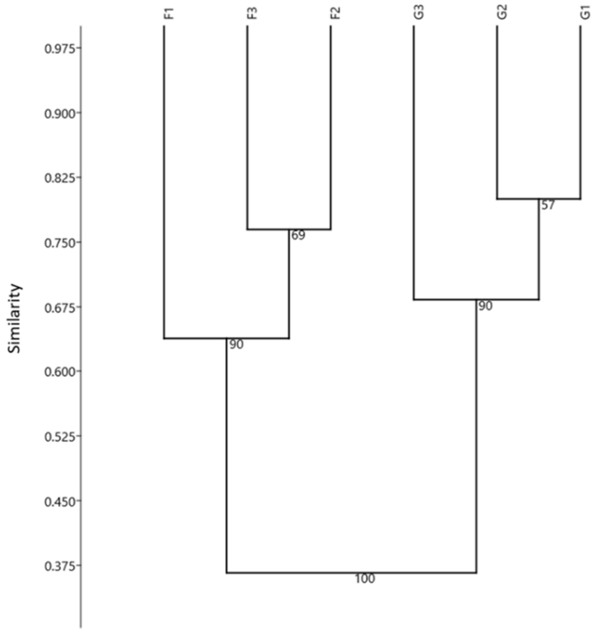

Figure S5. DGGE analysis of bacterial V6-V8 16S rDNA. Jaccard dendrogram of similarity. Samples were labeled as G and F for different caves followed by “1”, “2” or “3” that represent respectively, drip water, saturated sediment with drip water and unsaturated sediment samples

Text S1 – DGGE analysis

Methods

The V6-V8 region of the 16S rRNA was amplified in a thermocycler (Eppendorf) using 0.2 mM of each primer, 0.2 mM of dNTP, 0.03 U/μL of Taq DNA polymerase (Promega), 5.0 ng of DNA, 3 mM of MgCl2 and a 1× buffer. The PCR reaction was performed with the primers 984F (5′-AAC GCA AGA ACC TTA C-3′) and 1378R (5′-CGG TGT GTA CAA GGC CCG GGA ACG-3′) that amplify the 968 to 1401 nucleotide in *Escherichia coli* 16S rDNA [1] with a GC-clamp (5′-CGC CCG GGG CGC GCC CCG GGC GGG GCG GGG GCA CGG GGG G-3') [2] added to the 5′-end in the forward primer to avoid complete denaturation [18]. PCR was performed following these steps: 94 °C for 5 min; 35 cycles of 94 °C for 60 s, 60 °C for 90 s and 72 °C for 60 s; and a final extension at 72 °C for 5 min.

The *ndo* cluster III genes *nahAc* and *phnAc* were amplified with the primers NAPH-1F (5'-TGG CTT TTC YTS ACB CAT G-3') and NAPH-1R (5'-DGR CAT STC TTT TTC BAC-3') (Gomes et al. 2007) using the same concentration as described for the V6–V8 region. PCR was performed following these steps: 94 °C for 5 min; 35 cycles of 94 °C for 60 s, 52 °C for 90 s and 72 °C for 60 s; and a final extension at 72 °C for 5 min. A second-round PCR was performed in a semi-nested PCR approach using the NAPH-1F and NAPH-1R PCR product as template for a second reaction with NAPH-2F (5'-TAT CAC GGC TGG-3') and NAPH-2R (5'-ATS TCT TTT TCB AC-3') [3] primers with a GC-clamp [2] attached to the 5'-end of the NAPH-2F primer.

DGGE analysis of the 16S rDNA and *ndo* genes were performed using a DCode universal mutation detection system (Bio-Rad, Hercules, CA, USA). The GC-clamped DNA amplicons were applied to an 8% acrylamide:bisacrylamide gel (37.5:1) with a denaturing gradient of 30–55% for 16S rDNA and 30–70% for *ndo* genes. The run was performed in 1× TAE buffer at 60 °C with a constant voltage of 200 V for 4 h. The gel was then silver stained [4].

Two methods of cluster analysis based on the DGGE profiles were performed using a matrix composed of the presence or absence of bands: (1) non-metric multidimensional scaling (MDS) analysis; (2) UPGMA dendrogram using Dice's similarity coefficient in PAST 3.1 software (www.folk.uio.no/ohammer/past/).

Results

To investigate changes in the dominant bacterial community in drip water and sediment in two limestone caves in the semi-arid Bahia State, Brazil, a bacterial community fingerprint analysis was performed. The DGGE, which was used to compute the similarity dendrogram (Figure 1A), showed a separation between the caves. A non-metric MDS analysis also showed a similar differentiation between the caves (Figure 1B). GBP showed a similar profile among samples from drip water (G1) and saturated sediment (G2), and FFMP had a high similarity among the saturated (F2) and non-saturated (F3) sediment (Figure 1). This was corroborated by a non-metric MDS analysis that showed G1 and G2 nearest to each other, while F2 appeared slightly closer to F3 than F1. Bacterial communities from both caves presented a similarity of 37%.

Text S2 – Aromatic and *ndo* genes detection in other caves

Sediment samples were collected along two caves (Gruta do Lapão, a metasandstone cave, and Gruta de Manoel Ioiô, a limestone cave) from the entrance to approximately 600 m inside the caves. Both caves were without visible drip water. A total of four samples were collected in each cave and the procedures for quantification of aromatic compounds and detection of *ndo* genes were performed as previously described. Both aromatic and *ndo* genes were only detected in the entrance and in the first inner sample from these two caves (Table S2).

Text S3 – Diversity analysis

Methods

Good's coverage and diversity indices using ACE, Chao1, Jackknife, Shannon and Simpson were calculated in EZBioCloud database [5]. Those analyses were performed using all reads and normalized for 2000 reads.

Results

Diversity indexes were calculated for the reads of each sample and due to the variance in the number of reads, this analysis was performed with the complete set of reads and normalized (Table S1). In general, F1 and F2 samples presented higher values for ACE, Chao1 and Jackknife indices in the analysis with all reads; specifically, those samples presented a value 5 to 10 times higher than G1 and G2. However, once the analysis was normalized, F2 presented the lower value for ACE, Chao1 and Jackknife indexes, while F1 presented the higher value followed by G1 and G2 (Table S1). In both normalized and non-normalized analyses, sample F2 presented the lowest value for Shannon and highest value for Simpson indices, indicating lowest diversity in sample F2. For the Shannon index, sample F1 presented the highest value while G1 presented the highest value in normalized analysis. The Simpson index of sample G1 showed the lowest values in both analyses, indicating that this sample is the most diverse. According to Good's coverage, the complete reads analysis was able to access almost 100% for F1 and F2 samples (Table S1), while under normalized analysis the value for sample F1 dropped to 90% while the others remained at 98%. The operational taxonomic unit (OTU) count was higher in F1 and F2 samples with complete reads due to the higher number of sequences. In normalized analysis, sample F2 presented a considerably lower number of OTUs while F1 dropped to values close to the G1 and G2 samples.

References

1. Heuer, H.; Krsek, M.; Baker, P.; Smalla, K. Analysis of Actinomycete communities by specific amplification of genes encoding 16S rRNA and gel-electrophoretic separation in denaturing gradients. *Appl. Environ. Microbiol.* **1997**, *63*, 3233–3241.
2. Muyzer, G.; de Waal, E.C.; Uitierlinden, A.G. Profiling of complex microbial populations

by denaturing gradient gel electrophoresis analysis of polymerase chain reaction-amplified genes. *Appl. Environ. Microbiol.* **1993**, *59*, 695–700.

3. Gomes, N.C.M.; Borges, L.R.; Paranhos, R.; Pinto, F.N.; Krögerrecklenfort, E.; Mendonça-Hagler, L.C.S.; Smalla, K. Diversity of ndo genes in mangrove sediments exposed to different sources of polycyclic aromatic hydrocarbon pollution. *Appl. Environ. Microbiol.* **2007**, *73*, 7392–9, doi:10.1128/AEM.01099-07.

4. Marques, E.L.S.; Dias, J.C.T.; Silva, G.S.; Pirovani, C.P.; Rezende, R.P. Effect of organic matter enrichment on the fungal community in limestone cave sediments. *Genet. Mol. Res.* **2016**, *15*, doi:10.4238/gmr.15038611.

5. Yoon, S.-H.; Ha, S.-M.; Kwon, S.; Lim, J.; Kim, Y.; Seo, H.; Chun, J. Introducing EzBioCloud: a taxonomically united database of 16S rRNA gene sequences and whole-genome assemblies. *Int. J. Syst. Evol. Microbiol.* **2017**, *67*, 1613–1617, doi:10.1099/ijsem.0.001755.

Table S1 – Aromatic compounds detected along Gruta do Lapão and Gruta de Manoel Ioiô caves. Entrance sample (E) and first inner sample (1) from Gruta do Lapão (GL) and Gruta de Manoel Ioiô (GMI).

	**Aromatic compound (ng/g)**			
	Naphthalene	1-methylnaphthalene	phenanthrene	acenaphthene
**GL (E)**	1.78±0.62	-	1.36±0.51	2.18±0.74
**GL (1)**	1.39±0.53	-	9.24±0.42	1.03±0.55
**GMI (E)**	2.25±0.71	0.84±0.21	2.25±0.92	-
**GMI (1)**	1.89±0.40	-	4.96±10.44	-

Table S2 – Diversity indexes of bacterial community from cave samples.

	**OTU**		**Good's coverage**		**ACE**		**Chao1**		**Jackknife**		**Shannon**		**Simpson**	
	C	N	C (%)	N (%)	C	N	C	N	C	N	C	N	C	N
**F2**	501	18	99.8	98.8	649.2	31.3	586.7	24.4	665.0	28.0	0.47	0.34	0.864	0.877
**G1**	48	48	98.0	98.0	63.7	63.7	63.0	63.7	63.2	63.2	2.51	2.51	0.161	0.161
**G2**	63	50	98.7	98.0	84.0	66.9	70.2	60.9	80.0	66.0	2.39	2.39	0.194	0.194
**F1**	377	60	99.9	90.0	415.2	79.2	393.9	74.3	427.0	79.0	3.01	2.44	0.163	0.173

C – complete number of reads for each sample. N – Normalized number of reads (2000 reads)
